# Do Product Characteristics Affect Customers’ Participation in Virtual Brand Communities? An Empirical Study

**DOI:** 10.3389/fpsyg.2021.792706

**Published:** 2022-01-06

**Authors:** Zheng ShiYong, Li JiaYing, Wang HaiJian, Suad Dukhaykh, Wang Lei, Li BiQing, Peng Jie

**Affiliations:** ^1^Management School of Hainan University, Haikou, China; ^2^School of Business, Guilin University of Electronic Technology, Guilin, China; ^3^Management Department, College of Business, King Saud University, Riyadh, Saudi Arabia; ^4^School of Economics and Management, Wuhan University, Wuhan, China

**Keywords:** product characteristics, brand community, product complexity, product symbolism, product satisfaction

## Abstract

The virtual brand community has become an important marketing tool for companies. A successful brand community marketing strategy should attract a large number of consumers. Although past studies have revealed consumer motivations for participating in virtual brand communities, they fail to answer an important question: Why is it so easy for some virtual brand communities to attract users while others have such difficulty? In this study, product characteristics are hypothesized to be important factors that determine consumer motivation to participate in brand communities. Product characteristics (e.g., product complexity, product symbolism, and product satisfaction) can directly affect how actively consumers participate in brand communities. The results of questionnaires show that product complexity, product symbolism, and product satisfaction have a positive influence on consumers’ willingness to participate in brand communities. Notably, the duration of product use has a regulating effect on the influence of product satisfaction and product symbolism. A long period of product use weakens the influence of product satisfaction on consumers’ willingness to participate in brand communities. On the contrary, a long period of product use strengthens the influence of product symbolism on consumers’ willingness to participate in brand communities. This study enriches the literature on brand community participation and has implications for companies that aim to utilize brand communities for marketing.

## Introduction

A brand community is a social group of consumers who share the same interest in a brand without the limitation of geographical location (Lai and Liu, 2019; Bindewald et al., 2020). The development of social media has laid the foundation for the large-scale migration of brand communities to the Internet, giving rise to virtual brand communities. Research shows that in virtual brand communities, members share information, answer questions about products, and discuss their experiences with the products, which can strengthen their emotional ties to the community and the brand promise (Dash and Zhou, 2021). Therefore, companies are increasingly taking the initiative to establish virtual brand communities in the hope of communicating with consumers and strengthening the relationships between brands and consumers through these communities (Fini and Perry, 2020). Virtual brand communities established by companies include those on third-party social media, such as Renren.com, as well as those constructed by the companies independently, such as Meizu Community (Girija and Rani, 2020).

Although companies utilize brand communities with the intention of publicizing themselves, they are neither the main producers of the content in these communities nor the most important participants. The main players in virtual brand communities are the community users, and the success of a virtual brand community is dependent on consumers’ participation (Gruss et al., 2020). Thus, the key issue in community marketing is attracting and keeping community users to maintain the vitality of the community (Kitirattarkarn et al., 2019). Consumers’ participation in the community is directly influenced by their intrinsic motivations. Thus, many scholars have explained the participation of consumers in virtual brand communities from the perspective of their psychological motivations (Kumar et al., 2019; Lai and Liu, 2019). These studies are conducive to understanding the intrinsic motivations behind consumers’ participation in brand communities. However, studying motivation from a psychological aspect does not provide information that companies can directly use to manage their virtual brand communities (Mohammad et al., 2020). This study examines consumers’ participation in communities from the perspective of product characteristics. We propose that product characteristics can also affect consumers’ participation in these communities because they can influence their motivations to participate. For example, Xiaomi’s products clearly have more active community participation than those of Coolpad. Existing research fails to account for the effect of product characteristics on consumers’ participation in the associated brand community. From the perspective of brand management, linking product characteristics with consumers’ willingness to participate in communities can help scholars to understand why it is easy for some brands to utilize communities for marketing while it is hard for others.

According to previous studies on this subject (Toubia et al., 2021), the motivations that drive consumers to participate in virtual brand communities can be divided into function-driven factors, society-driven factors, and emotion-driven factors (Windasari and Visita, 2019). This study examines the influence of major product characteristics driven by three kinds of factors—product complexity, product symbolism, and product satisfaction—on consumers’ willingness to continuously participate in virtual brand communities. The results suggest that the three characteristics have positive effects on consumers’ participation in virtual brand communities. Given the fact that continuous community participation is a dynamic process, this study also explores the role of the duration of product use in regulating the relationships between product symbolism and continuous community participation and between product satisfaction and continuous community participation. The results show that the duration of product use weakens the relationship between product satisfaction and consumers’ willingness to continuously participate in communities, whereas it strengthens the relationship between product symbolism and consumers’ willingness to continuously participate in communities.

The theoretical contributions of this study are as follows: (1) The results show that product characteristics are important factors that determine whether consumers are willing to continuously participate in communities. Unlike past research, which either studied the psychological aspects of consumer motivations to participate in brand communities or discussed the sociological aspects of community performance of virtual brand communities, this study reveals the influence of product characteristics on brand community participation, enriching the literature on brand community participation. (2) This study reveals that brand community participation and the consumer–brand relationship interact as both causes and effects. Past research has found that community participation can promote the consumer–brand relationship (e.g., brand loyalty and brand promise), but this study also reveals that product satisfaction can promote brand community participation, which indicates that satisfied consumers are more likely to engage in social media marketing. (3) Our research suggests that the motivations of consumers participating in brand communities continue to change. We find that the duration of product use is connected with brand community participation. As the duration of use increases, the relationship between product satisfaction and community participation is weakened, but the relationship between product symbolism and community participation is strengthened. This indicates that as consumers become familiar with products, their focus shifts to the symbol that the products represent. This research can help companies to tailor the focus of their brand communities according to the characteristics of their product in order to promote user participation.

## Theory Review and Hypotheses

### Brand Community

A brand community is the product of the combined influences of a market-oriented economy, consumerism, and mass media, and its connotations are still expanding. The initial purpose of establishing brands was for companies to differentiate themselves from others so as to increase their competitiveness. With the help of widespread mass media, companies attract consumers by highlighting their brand image and brand connotations to imply that their products possess not only physical qualities but also spiritual qualities. Brand communities are groups of consumers who gather out of their love for particular brands; participants form specific connections, rituals, and traditions among themselves and show a sense of responsibility toward the community (Zhang and Xue, 2019). With the development of communication technologies, people who are far away from each other can connect because of their shared spiritual bonds. In such cases, communities are no longer limited to certain areas. The meaning of “community” is expanding. At present, brand communities are predominantly based on social media. In the era of social media, the brand community has become an effective channel through which companies can establish strong relationships with consumers (Barlas et al., 2020), as it promotes brand loyalty (del Rocío Bonilla et al., 2020) and is a platform for feedback that can be utilized for product development (Gaworski et al., 2021). Therefore, brand communities always reflect consumers’ specific feelings toward the associated brands. Consumers will spontaneously match the experiences and images conveyed by the brands with their own values to further strengthen their connection with the brands.

### Motivations of Consumers’ Participation in Brand Communities

Willingness to engage is the likelihood that users will be willing to respond to a company’s activities once they have entered a virtual brand community. Existing research shows that user engagement is fundamental to the long-term survival and growth of user innovation communities (Kumar et al., 2019; Lai and Liu, 2019). Therefore, in order to improve the success and continued survival of user innovation communities, managers have to understand the motivations for user engagement in communities and then take appropriate measures to increase the activity of user innovation communities, maintain the continuity of community user engagement, and provide assistance to companies’ product innovation (Windasari and Visita, 2019).

In this part, we identify product characteristics related to consumers’ participation in brand communities. With the development of the economy and the improvement of people’s living standards, modern consumer requirements have transcended physical qualities. Their attention has shifted from the functions of the products to the social significance and emotional satisfaction that they derive from them. Studies on marketing suggest that consumers not only become dependent on the products that they buy but also become fixated on the brands. Some researchers argue that this fixation can strengthen consumers’ willingness to participate in brand communities (Goli et al., 2021); in other words, if consumers recognize a brand, they experience a high level of consistency, are willing to seek channels to maintain a long-term relationship with the brand and more actively participate in the brand community. To be more specific, consumers differ in their motivations to participate because of their varying requirements for brand communities.

Based on past research, this study divides consumer motivations to participate in brand communities into three types (Han et al., 2019; Akram et al., 2021): function-driven factors, society-driven factors, and emotion-driven factors. Function-driven factors drive people to seek information (Kuang et al., 2019), society-driven factors involve people’s desire to express themselves and seek friendship, and emotion-driven factors refer to people’s instincts to share their emotions (Malthouse et al., 2016; Jayaraman and Abirami, 2020). Although there might be other factors, we focus on these three types of factors according to past research.

### Function-Driven Factors: Product Complexity

The Internet is one of the means by which consumers acquire information about products, brands, and services. Virtual communities can provide a wide range of benefits (Genta et al., 2018), such as supporting the development of interpersonal relationships, providing people with a sense of companionship, and belonging and encouraging people to discuss and share knowledge. They allow members to acquire information, instantly share their opinions, and provide social and emotional support, enabling group activities (e.g., software development). One of the major consumer motivations for engaging in brand communities is to acquire product information and ask questions about products. Consumers who visit a brand community can obtain information by browsing through its content, which can promote their participation (Hvam et al., 2020).

Product complexity can strengthen consumer motivation to acquire information, so it might be a factor that drives consumers to participate in brand communities. The dictionary defines the word “complexity” as something composed of interconnected parts. The complexity of products can exert profound effects on consumers’ purchasing and use behaviors. Some scholars define product complexity as the difficulty that consumers have in understanding or using the products (Rahmati et al., 2021). If consumers have to go through a series of settings and procedures before using a product, the product is considered complex. When consumers perceive a product as complex, uncertainty might arise due to their difficulty in understanding it. As the probability of negative and uncertain results increases, the consumer’s perceived risk also increases. Likewise, when a complex product has too many attributes, information collection and attribute comparison will become difficult (Berger and Heath, 2007; Zhang and Xue, 2019). Some researchers claim that as a product becomes more complex, consumers are more likely to adopt heuristic thinking and process the information selectively, and thus, their decisions become less effective (Tsai and Bagozzi, 2014). These researchers argue that consumers’ decision making is influenced by product complexity. Some studies have indicated that for complex products, information recommendation exerts more significant effects on consumers, which demonstrates that product complexity might increase their motivation to search for information (Marzocchi and Morandin, 2013; Akram et al., 2021).

### Society-Driven Factors: Product Symbolism

Brand communities are groups of consumers who share the same preference for certain brands. Recognition is a key factor that drives consumers to participate in brand communities (Belk et al., 2021). As a type of society, communities encourage members to interact, help each other, and exchange information. In addition, research has also found that a key driving factor of community participation is the social benefit that the communities provide to consumers (in other words, the friendship among community members; Solomon and Susan, 2021). Other researchers have discovered that trust and reciprocity are major consumer motivations for sharing content (Akram et al., 2018). Perceiving the social values of communities will help to promote consumer participation in brand communities (Akram et al., 2020).

As suggested by product value theory, symbolic value is part of the product value. According to symbolic interactionism, a product is endowed with symbolic meaning, and people will appreciate its connotations through learning (Reed and Miller, 2020). Appreciation of symbolism is a common cultural phenomenon because people want to express themselves through products and study others through the products that they possess (Toubia et al., 2021). Consumers see their possessions as an extension of themselves. The products are not only physical objects but also an embodiment of subjective symbolism (Chen et al., 2021). Consumers want to buy products with brand images that are consistent with their own, and they use symbolic products to demonstrate the social groups to which they belong. As indicated by sociological theories, member similarity is one of the key factors in the formation of groups. Thus, the more symbolic the products, the greater the difference between product users and non-users and the greater the similarity among users.

This can help product users to find people who are similar to themselves and acquire social experience and social values.

### Emotion-Driven Factors: Product Satisfaction

In essence, a brand is a concept with emotional factors. Researchers have pointed out that brand communities are groups of consumers who love and admire certain brands. Thus, emotional factors are key in driving consumers to join virtual brand communities (Changchit and Tim, 2020).

Emotional factors allow consumers to establish stable relationships with brands. Product satisfaction might be an important emotional factor that drives consumers to participate in brand communities, as it renders consumers fixated on the brand (Li and Chen, 2021). It is an important concept in marketing theories and practices and is closely related to changes in consumers’ attitudes, repeat purchases, and brand loyalty (Ghosh, 2020). Consumer satisfaction can also enhance the effects of advertisements and even increase human capital performance (Hansen et al., 2019). Some studies have revealed that satisfied consumers are more willing to pay a premium for products (Mirhoseini et al., 2021) and are less sensitive to prices. Consumer satisfaction can be divided into four aspects: consumer, employees, efficiency, and overall performance. There are few studies on consumer satisfaction from the perspectives of efficiency and employees. When consumers are satisfied, they will be more loyal to the brands and more willing to publicize them, and thus, the brands will lose fewer consumers (Tang et al., 2017; Akram et al., 2021; Noranee and Abdul, 2021). However, whether consumer satisfaction affects the communities of the brands of the purchased products has not been studied.

## Model Framework and Hypotheses


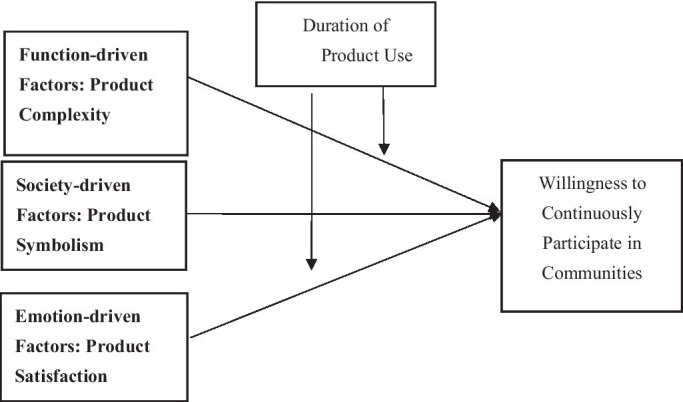


### Product Complexity and Consumers’ Willingness to Participate in Communities

Searching for information about products or brands is an important motivation for joining virtual brand communities (Christakis and Fowler, 2017). Answers in communities about products are sometimes more important than answers from companies: (1) Firstly, as companies have insufficient online customer service staff, it always takes a long time for consumers’ questions to be answered. In comparison, the recipients of information requests in virtual communities are all community members. Thus, they are more efficient in answering questions. (2) Most answers provided by companies’ customer service personnel are standardized. Thus, they are far from sufficient to satisfy consumers’ demand for personalized responses and specific solutions. In contrast, community members can provide more detailed information from different aspects.

In the wake of scientific, economic, and social development, products are becoming increasingly complex, which makes it increasingly difficult for consumers to use them. Some researchers have found that companies need to provide customer education activities to help consumers to learn to use the products on their own and enhance their loyalty. Therefore, the more complex the products, the more knowledge that consumers require. In the era of social media, brand communities are the main channels through which consumers acquire information about products. On the one hand, consumers can directly acquire knowledge about products by reading the content in communities. On the other hand, consumers can view feedback from other consumers through their interactions. The more complex the products, the more information that consumers need, and thus, the more dependent they become on virtual communities. In other words, if the community can provide useful information to consumers, then they are more likely to think it is useful and are more willing to continuously participate in the community. On this basis, we propose the following hypothesis:

*H1:* The higher the product complexity, the stronger consumers’ willingness to participate in the virtual brand community.

### Product Symbolism and Consumers’ Willingness to Participate in Communities

There are two aspects of social recognition of an individual: the individual perception of his or her identity in the community and the importance of emotions and assessments of this identity (Butler et al., 2018). Inspired by this concept, some scholars claim that social recognition includes several components. Cognition component: The consumer’s belief that he or she belongs to a certain group of people, which is called self-classification (Yuan et al., 2017); Assessment component: The consumer’s positive or negative values of identity in the group, which is called group dignity; and Emotion component: The consumer’s emotional participation in the group, which is called emotional promise. According to this theory, a more symbolic product has more symbolic meaning, and thus, it is more able to differentiate its users from other social groups and serve as a symbol of the consumer group associated with the brand (Boyd, 2017). In such cases, the brand community will endow the product with strong social group implications, which can increase consumers’ willingness to participate in the community. Therefore, we propose the following hypothesis:

*H2:* The stronger the product symbolism, the stronger consumers’ willingness to participate in the virtual brand community.

### Product Satisfaction and Consumers’ Willingness to Participate in Communities

Some researchers suggest that consumer satisfaction can establish an emotional connection between consumers and brands (Tang et al., 2017). Other researchers argue that consumer satisfaction can promote customer engagement (Changchit and Tim, 2020). Consumers’ willingness to help publicize products has a U-shaped relationship with consumer satisfaction (Noranee and Abdul, 2021); in other words, extremely satisfied and extremely dissatisfied consumers are most likely to publicize products. Extreme emotions can easily evoke consumers’ willingness to publicize products (Changchit and Tim, 2020). However, it should be noted that cases of extreme consumer dissatisfaction are rare and mostly occur in experiments. In practice, most consumers are either satisfied or extremely satisfied. Thus, we do not examine this U-shaped relationship (Bandura, 2017). Therefore, consumers who are very satisfied are more willing to participate in discussions over the reputation of the products in virtual brand communities and are more willing to tell other consumers about their experience with the brands and spread their knowledge about the products among other people. Thus, we propose the following hypothesis:

*H3:* The higher the consumer satisfaction, the higher the consumers’ willingness to continuously participate in the virtual brand community.

### The Regulating Effect of Duration of Product Use

The duration of product use has a regulating effect on the influence of product symbolism on consumers’ willingness to continuously participate in communities. According to consumer self-concept theory, consumers tend to see their possessions as an extension of themselves (Black and Veloutsou, 2017). The more symbolic the products, the more inclined consumers are to use the products to define themselves and spread this form of self-concept to others (Zhang et al., 2016). For products with strong symbolism, consumers form a special relationship with the brand and develop a psychological self-brand connection over a long period of use (Reed and Miller, 2020). In such cases, consumers are more willing to continuously participate in brand communities. Based on this deduction, we propose the following hypothesis:

*H4:* The longer the duration of product use, the stronger the consumers’ willingness to participate in the brand community.

The duration of product use also has a regulating effect on the influence of product satisfaction on consumers’ willingness to continuously participate in communities. In previous research, subjects were asked about their degree of satisfaction with a theft-proof watch and their willingness to purchase the product. Two weeks later, they were asked the same questions again (Toubia et al., 2021). The results suggest that the degree of satisfaction can be used to predict the willingness to purchase the first time but not the second time, which indicates that the correlation between satisfaction and willingness might decline with time. We deduce that consumers’ degree of satisfaction in the early stage of product use can be used to predict their willingness to continuously participate in the brand community. However, consumers become familiar with products that they have used for a long time, and thus, their willingness to participate in communities declines. Based on this deduction, we propose the following hypothesis:

*H5:* The longer the duration of product use, the weaker the consumers’ willingness to participate in the brand community.

## Data Analysis and Examination

Using questionnaires, data were collected from 427 consumers from brand communities of four types of products: automobile, computer, mobile phone, and sneakers. The questionnaires include three parts: (1) Identification of the brands of the products used by consumers, the duration of use, and their assessment of the product characteristics (product satisfaction, product complexity, and product symbolism); (2) Investigation of consumers’ willingness to participate in brand communities based on the brand communities that they are in; and (3) Collection of demographic information from the interviewees. To encourage community members to complete the questionnaires, we gave 5 yuan to each interviewee and provided them with a chance to win a 50-yuan raffle.

We applied the following measures to ensure the quality of the questionnaires: (1) Questionnaires from the same IP address and the same computer were considered invalid; (2) Questionnaires that were finished within 30 s were excluded; and (3) In the end, 375 of the samples met the criteria. The statistical characteristics of valid samples are shown in Table 3. The results show that 114 consumers had used the products for less than 6 months, accounting for 30.4% of the samples; this was encoded as 0, which represents a short duration of use. A total of 106 consumers had used the products for 6–12 months, accounting for 28.3% of the samples; this was encoded as 1, which represents a medium duration of use. Finally, 155 consumers had used the products for more than 12 months, accounting for 41.3% of the samples; this was encoded as 2, which represents a long duration of use.

**Table 1:** Demographic data of the samples.

Demographic variables	Type	Number	Proportion
Gender	Male	266	70.9%
	Female	109	29.1%
Age	Less than 25	216	57.6%
	25–34	118	31.5%
	More than 35	41	10.9%
Income	Less than 1,000 yuan	87	23.2%
	1,000–2000 yuan	61	16.3%
	2000–3,000 yuan	80	21.3%
	3,000–4,000 yuan	77	20.5%
	4,000–5,000 yuan	45	12.0%
	More than 5,000 yuan	25	6.7%
Educational Background	Junior College and High School and Lower	110	29.3%
	Graduate	232	61.9%
	Postgraduate and Higher	33	8.8%

### Construct Measurement and Examination

This study involves four constructs: product complexity, product symbolism, product satisfaction, and willingness to continuously participate in communities. The scale for product symbolism is from (White and Argo 2014), the scale for product complexity is from (Lovett et al., 2013), the scale for product satisfaction is from (Lovett et al., 2013), and the items about willingness to continuously participate in communities are from (Algesheimer et al., 2005). All constructs were measured using a 7-point Likert scale, where 7 indicates totally agree, and 1 indicates totally disagree (Windasari and Visita, 2019; Chen et al., 2021).

Reliability and validity tests. Reliability tests include internal consistency reliability and composite reliability tests. As shown in Table 2, the Cronbach’s α values of all constructs are higher than 0.838, indicating that all constructs have high internal consistency. The composite reliability (CR) values of all constructs are higher than 0.880, indicating that all constructs have high composite reliability. Confirmatory factor analysis was applied to test convergent validity. The factor loadings of all observation items are higher than 0.75. Furthermore, the results of the fit index of the measurement model show that *χ*^2^(38) = 106.854, *χ*^2^/df = 2.811, *p* < 0.001, RMSEA = 0.079, CFI = 0.965, NFI = 0.951, IFI = 0.965, and GFI = 0.943, which indicate that convergent validity is high.

**Table 2:** Latent variables reliability and validity tests.

Construct	Measured Item	Factor Loading	Cronbachα
Product Complexity	I need to learn how to use this brand well.	0.848	0.850
	I need to spend some time on exploration to solve problems that occur when I use the product.	0.863	
	I need to spend some time to fully understand the product when I use it.	0.879	
Product Symbolism	This brand can reflect some characteristics of its users.	0.851	0.838
	This brand matches some characteristics of its users.	0.887	
	This brand can stand for a group of consumers.	0.764	
Product Satisfaction	Generally, I am satisfied with the product.	0.910	0.900
	I am pleased when using this product.	0.901	
	This product lives up to my expectation.	0.833	
Willingness to Continuously Participate	I will continue my participation in this brand community.	0.924	0.930
	I will participate in discussions in this community later.	0.920	

Table 3 reports the composite reliability and average variance extracted (AVE) of the latent variables. With the factor loadings from confirmatory factor analysis, we can calculate the composite reliability of the latent variables, which are higher than 0.873, above the reference value of 0.7. In addition, their average variance extracted is higher than 0.698, above the threshold value recommended by scholars. These indicators demonstrate that the scale adopted by this research has good construct reliability and validity. Our results reveal that the AVE values are all higher than the square of the correlation coefficient between the different variables, which indicates that the variables have good discriminant validity. In conclusion, the data in this research have good reliability and validity and can be used for further tests and analysis.

**Table 3:** Latent variable correlation matrix.

Variable	1	2	3	4
Product Complexity	1			
Product Symbolism	0.327^*^	1		
Product Satisfaction	0.191^**^	0.397^**^	1	
Willingness to Continuously Participate	0.271^**^	0.333^**^	0.436^***^	1
Average Value	4.000	4.813	5.550	4.721
Standard Deviation	1.135	1.336	1.178	1.692
Composite Reliability	0.897	0.873	0.913	0.919
Average Variance Extracted (AVE)	0.745	0.698	0.777	0.850

* *indicates 0.1 level of statistical significance*;

** *indicates 0.05 level of statistical significance*;

*** *indicates 0.01 level of statistical significance (the same meanings apply hereinafter)*.

### Common Method Variance Analysis

To prevent the common method variance caused by questionnaires filled out by the same person, discriminative reverse expressions were added to the observation items of some constructs. With these discriminative reverse expressions, we can exclude self-contradictory samples. Furthermore, the data were examined using two methods. The first method was Harman’s one-factor test, which was used to conduct exploratory factor analysis on all observation items of the constructs. If the first factor’s variance before rotation accounts for over 50%, it means that the common method has high variance. The results in this research were calculated using SPSS19.0. The variance of the first factor accounts for 39.43%, which is below 50%. This indicates that the common method variance of the data is within a good range. The second method involved examining the correlation coefficients between the constructs. If it is higher than 0.9, it means that there is high common method variance. A result less than 0.9 is acceptable. From Table 3, it can be seen that the highest correlation coefficient between the constructs is 0.436, much less than 0.9. Therefore, the data in this research are reliable. The results of these two methods indicate that the data in this research do not have a serious common method variance problem. Thus, they can be used for the analysis.

### Hypothesis Tests and Results

Hierarchical stepwise regression was applied to examine the hypotheses, and the results are shown in Table 4. Model 1 is the regression result for the influence of the main variables and control variables on dependent variables. In Model 2, the duration of product use is included as an independent variable to test its regulating effect. According to the data from Model 1, the coefficient of the influence of product complexity on consumers’ willingness to continuously participate in communities is significant (*β* = 0.124**), which supports H1. In other words, when consumers perceive a product as complex, they are more willing to continue participating in the community. The influence of product symbolism on consumers’ willingness to continuously participate in communities is significant (Model 1: *β* = 0. 173***; Model 2: *β* = 0.159***), which supports H2. This means that consumers are willing to interact with other consumers who share the same preference for certain brands because of their symbolic value. The influence of product satisfaction on consumers’ willingness to continuously participate in communities is significant (Model 1: *β* = 0. 307***; Model 2: *β* = 0.426***), which supports H3. Past research has found that product satisfaction can generate a positive reputation among consumers, prevent the loss of consumers, and increase the brand loyalty of consumers. In this research, however, we discovered that product satisfaction can also affect consumers’ participation in brand communities, which is a dependent variable that should not be ignored. All companies want to conduct community marketing, but they might fail to realize that community marketing is more effective for satisfied consumers (compared to consumers with a low or medium degree of satisfaction).

**Table 4:** Results of model tests.

Variable	Dependent variable: willingness to continuously participate in communities	
	Model 1	Model 2
Product Complexity	0.124^**^	0.201^**^
Product Symbolism	0.173^***^	0.159^***^
Product Satisfaction	0.307^***^	0.426^***^
Duration of Product Use ^*^ Product Symbolism		0.246^***^
Duration of Product Use ^*^ Product Satisfaction		−0.159^**^
Duration of Product Use	−0.020	0.663^**^
Age	0.066	0.068
Gender	−0.074^*^	0.078*
Income	0.165^***^	0.173^***^
Educational Background	−0.178^***^	−0.165^***^
Intercept term	1.499^**^	−0.876
R-squared	0.319	0.336
Adjusted R-squared	0.304	0.318
*F*-value	21.500^***^	18.440^***^
N, df	366,8	364,10

* *indicates 0.1 level of statistically significance*;

** *indicates 0.05 level of statistically significance*;

*** *indicates 0.01 level of statistical significance (similarly hereinafter).*

We arrived at an interesting conclusion on the regulating effect of the duration of product use. The influence of the duration of product use on consumers’ willingness to participate in communities is positively significant (*β* = 0.246***), which supports H4. This means that for products with a high degree of symbolism, a long duration of use can help to develop a psychological bond between the consumers and the brands, and thus, consumers will have a strong desire to strengthen their relationship with the brands. Finally, as we expected, the duration of product use has a negative regulating effect on the influence of product satisfaction on consumers’ willingness to participate in communities (*β* = −0.159***), which supports H5. This means that the influence of product satisfaction on consumers’ willingness to participate in communities will decline over time as consumers use the product. This is consistent with the conclusion drawn in past research that the effectiveness of using product satisfaction to predict consumer behaviors declines with the passage of time. In addition, it is also discovered that income has a positive correlation with consumers’ willingness to continuously participate in virtual communities, whereas educational background has a negative correlation. Pragmatists might join communities when they have just purchased products but will leave shortly thereafter. Age does not have a significant influence on consumers’ willingness to continuously participate in virtual communities.

## Discussion and Conclusion

This paper empirically examines the factors that contribute to consumer engagement with brand communities based on a product perspective. Previous research has argued that consumers tend to prefer simple, easy-to-use products, and therefore, high product complexity reduces the user experience and therefore the willingness to engage with the product. However, this study shows that product complexity is an important driver of consumer engagement with brand communities. As product complexity increases, consumers need to acquire a great deal of knowledge in order to better use the product, which leads to brands with more product complexity being more likely to achieve community marketing success. This suggests that consumers’ own experience and knowledge may influence their choice of products and subsequent brand community engagement behavior. We can pay further attention to this issue in future research. In addition, this study found that consumer product satisfaction can increase consumers’ willingness to engage with their communities. Consumer satisfaction remains important in the age of social media, and consumer satisfaction strengthens consumers’ willingness to continue building relationships with brands, which is consistent with the findings of previous research related to consumer satisfaction and user engagement. This is in line with previous research on consumer satisfaction and user engagement, where the higher the consumer satisfaction, the stronger the user’s willingness to engage.

Finally, this paper’s research found that product symbolism has a significant positive impact on consumer participation in online communities. This may be due to the fact that despite the fact that online communities are a stranger’s environment, consumers still have a strong willingness to express themselves through symbolic consumption and seek social support. Previous studies, however, have paid less attention to the impact of symbolic features of products on user engagement behavior, which is one of the important contributions of this study. Previous research related to consumer loyalty points out that the longer a product is used, the more conducive it is to the formation of brand loyalty. Brand loyalty, in turn, promotes continued user engagement. This study finds that the previous research is invalid in certain scenarios. On the one hand, this study finds that product usage time drives changes in consumers’ ongoing engagement with brand communities to some extent, a finding that is somewhat consistent with findings related to consumer loyalty. On the other hand, this study found that when product satisfaction was a driver of consumer engagement in brand communities, this driver diminished as product usage time increased; this is inconsistent with previous findings. Furthermore, the relationship between product symbolism and willingness to participate in the community became stronger as time of use increased.

## Theoretical Contributions

First, this study is the first to connect product (brand) characteristics with consumers’ willingness to participate in virtual brand communities. It explores the role of the brand in driving consumers to participate in virtual brand communities and enriches the literature on consumers’ participation in these communities. Many studies have investigated the motivations of consumers to participate in virtual brand communities, but most of them have been carried out purely from the perspective of consumer motivations (Xu, 2020). One of the theoretical contributions of this study is that it analyzes the influence of product characteristics on consumers’ participation in communities, providing explanations for why the results of community marketing vary so much among different brands.

Secondly, this study enriches the literature on the influence of product satisfaction. Past research has proven that product satisfaction can increase the brand loyalty of consumers so that they voluntarily contribute to the brand reputation. This study, however, proves that product satisfaction can promote consumers’ willingness to participate in brand communities. In this study, the influence of product satisfaction is extended to consumers’ participation on social media. As the results suggest, satisfied customers are more likely to engage in social media marketing. Past research has revealed that community participation can improve the quality of the relationship between consumers and brands (e.g., brand loyalty and brand promise), but this study also reveals that product satisfaction can promote brand community participation. Therefore, brand community participation and the consumer–brand relationship interact as both causes and effects. Their relationship is not a one-way street, as suggested in past research. Therefore, this research contributes to the literature on the association between brand relationship and brand community participation.

Finally, this study also reveals dynamic factors that affect consumers’ participation in brand communities. As we observed, the duration of product use is connected with brand community participation. When consumers use products for a long time, the relationship between product satisfaction and community participation weakens, but the relationship between product symbolism and community participation strengthens. Therefore, consumers’ relationship with communities is constantly changing and is closely related to the duration of product use. With this discovery, scholars will be able to observe consumers’ community participation more dynamically.

## Implications For Management

This study provides some implications for companies seeking to establish virtual brand communities.

Firstly, past research has discovered that more than half of virtual brand communities have failed due to the lack of content and interaction. Then, we should ask a question: What kind of brand is suitable for a brand community, or will brand characteristics affect consumers’ willingness to participate in brand communities? The research in this study suggests that products with a high degree of symbolism and complexity are suitable for community marketing because community marketing can best satisfy consumer demand for social interaction and information.

Secondly, consumer satisfaction is still very important in the era of social media. Consumer satisfaction can strengthen consumers’ desire to establish relationships with brands and increase their brand loyalty. Therefore, the success of community marketing should not be focused solely on its operation but also on consumers’ product satisfaction. This requires companies to consider whether their products are suitable for a brand community from a strategic perspective and how to conduct community marketing from the perspective of the product. Only in this way can they increase the probability of successful community marketing.

Thirdly, different brands can apply different strategies to establish their brand communities. For example, for brands with a low degree of complexity (e.g., Coca-Cola), consumers participate in their online communities not because they seek useful information but because of the unique symbols that these brands represent (e.g., Coca-Cola represents energy and feelings of refreshment). This symbolic value can help to drive loyal customers to participate in the associated brand communities. This means that consumer requirements for brand communities depend on product characteristics. Brands whose products feature specific functions and have a high degree of complexity can highlight the functional value (e.g., useful information and problem solving) of their brand communities. On the contrary, brands with strong symbolism should emphasize the similarity of their values to those of consumers and resonate with them so as to encourage their participation in brand communities.

Lastly, managers of brand communities should realize that consumer motivations to participate in brand communities are dynamic. Consumers gain more knowledge about products as they use them, and thus, the influence of product symbolism on the willingness to participate in communities is strengthened over time, but the influence of product satisfaction is weakened. Therefore, for new users, brand communities should provide them with content about information and functions to make the community more attractive to them. For experienced consumers, the communities should emphasize intangible values of the products, such as their symbolic value, to increase brand recognition. Moreover, the passage of time has a negative regulating effect on the influence of product satisfaction on consumers’ willingness to participate. Hence, to liven up virtual brand communities, companies should continuously attract new users to participate.

## Limitations and Future Studies

This paper also has certain limitations. Firstly, the source of the data in this study was a questionnaire and the findings of the study would be more convincing in future studies if secondary data could be used to validate the findings. For example, crawlers were written using programming languages, such as python to collect behavioral data from users in various brand communities. However, this requires a certain technical threshold. Secondly, the products involved in this study are more technologically complex (e.g., cars, computers, and mobile phones), while some studies have pointed out that there is a difference in the choice preferences of men and women on more technologically advanced products. Female consumers are more concerned with the appearance of the product, while male consumers are more concerned with the performance of the product. This has therefore led to the sample participating in this study being mostly male users. And in future studies, we can also expand to products that female consumers care about (e.g., cosmetics) to further test the findings of this study. Thirdly, this study discusses the influence of brand characteristics on consumers’ participation in virtual brand communities based on consumer motivation theory, but it fails to reveal the rationale behind the influence of each brand characteristic on consumer motivation to participate. Future studies can explore this issue with both experiments and surveys. Lastly, although this study discusses the influence of three major product characteristics on consumers’ participation in brand communities, the influence of other brand or product characteristics (e.g., brand personality) on consumers’ participation in virtual brand communities still remains to be studied.

## Data Availability Statement

The raw data supporting the conclusions of this article will be made available by the authors, without undue reservation.

## Author Contributions

ZS is responsible for article writing and model building, LJ for literature translation, WH for building research ideas and models, WL for data collection, LB for data analysis, and SD and PJ for revising the paper critically for important intellectual content. All authors contributed to the article and approved the submitted version.

## Conflict of Interest

The authors declare that the research was conducted in the absence of any commercial or financial relationships that could be construed as a potential conflict of interest.

## Publisher’s Note

All claims expressed in this article are solely those of the authors and do not necessarily represent those of their affiliated organizations, or those of the publisher, the editors and the reviewers. Any product that may be evaluated in this article, or claim that may be made by its manufacturer, is not guaranteed or endorsed by the publisher.
